# Analysis of *Schistosomiasis haematobium* Infection Prevalence and Intensity in Chikhwawa, Malawi: An Application of a Two Part Model

**DOI:** 10.1371/journal.pntd.0002131

**Published:** 2013-03-21

**Authors:** Michael G. Chipeta, Bagrey Ngwira, Lawrence N. Kazembe

**Affiliations:** 1 Malawi Liverpool-Wellcome Trust Clinical Research Programme, Blantyre, Malawi; 2 Community Health Department, University of Malawi, College of Medicine, Blantyre, Malawi; 3 Statistics Department, University of Namibia, Windhoek, Namibia; London School of Hygiene & Tropical Medicine, United Kingdom

## Abstract

**Background:**

Urinary Schistosomiasis infection, a common cause of morbidity especially among children in less developed countries, is measured by the number of eggs per urine. Typically a large proportion of individuals are non-egg excretors, leading to a large number of zeros. Control strategies require better understanding of its epidemiology, hence appropriate methods to model infection prevalence and intensity are crucial, particularly if such methods add value to targeted implementation of interventions.

**Methods:**

We consider data that were collected in a cluster randomized study in 2004 in Chikhwawa district, Malawi, where eighteen (18) villages were selected and randomised to intervention and control arms. We developed a two-part model, with one part for analysis of infection prevalence and the other to model infection intensity. In both parts of the model we adjusted for age, sex, education level, treatment arm, occupation, and poly-parasitism. We also assessed for spatial correlation in the model residual using variogram analysis and mapped the spatial variation in risk. The model was fitted using maximum likelihood estimation.

**Results and discussion:**

The study had a total of 1642 participants with mean age of 32.4 (Standard deviation: 22.8), of which 55.4 % were female. Schistosomiasis prevalence was 14.2 %, with a large proportion of individuals (85.8 %) being non-egg excretors, hence zero-inflated data. Our findings showed that *S. haematobium* was highly localized even after adjusting for risk factors. Prevalence of infection was low in males as compared to females across all the age ranges. *S. haematobium* infection increased with presence of co-infection with other parasite infection. Infection intensity was highly associated with age; with highest intensity in school-aged children (6 to 15 years). Fishing and working in gardens along the Shire River were potential risk factors for *S. haematobium* infection intensity. Intervention reduced both infection intensity and prevalence in the intervention arm as compared to control arm. Farmers had high infection intensity as compared to non farmers, despite the fact that being a farmer did not show any significant association with probability of infection.

These results evidently indicate that infection prevalence and intensity are associated with risk factors differently, suggesting a non-singular epidemiological setting. The dominance of agricultural, socio-economic and demographic factors in determining *S. haematobium* infection and intensity suggest that disease transmission and control strategies should continue centring on improving socio-economic status, environmental modifications to control *S. haematobium* intermediate host snails and mass drug administration, which may be more promising approaches to disease control in high intensity and prevalence settings.

## Author Summary

Schistosomiasis is one of the great causes of morbidity among school aged children in the tropical region and Sub Saharan Africa in particular. It's mainly transmitted through contact with water infested with intermediate host snail Cercariae. Currently, over 200 million people are estimated to be infected in SSA alone. Here, we used robust and contemporary statistical methods in a two part application to analyse risk factors for *S. haematobium* infection intensity and prevalence. We found that *S. haematobium* was more common in younger children as compared to older children, thus making the infection and prevalence age dependent. We also found that mass chemotherapy reduced both infection prevalence and intensity. We found that dominance of agricultural, socio-economic and demographic factors in determining *S. haematobium* infection risk in the villages carries important implications for disease surveillance and control strategies. Therefore disease transmission and control strategies centered on improving strategies involving socio-economic status, environmental modifications to control *S. haematobium* intermediate host snails and mass drug administration may be more promising approaches to disease control in high intensity and prevalence settings.

## Introduction

According to [1], Schistosomiasis infections affect an estimated 779 million people, with consequences in health nutritional and educational development of infected individuals [2]. The disease causes an annual loss of 4.5 million disability-adjusted-lifeyears (DALYs) [3]. In SSA alone, 207 million individuals are estimated to be infected with Schistosomiasis: *S.haematobium* and *S.mansoni* [1]. *S.haematobium* is reported to be endemic in 53 countries in the Middle east and most of the African continent including islands of Madagascar and Mauritius [4], whereas *S.mansoni* is mostly endemic in sub-Saharan Africa [4]. Schistosomiasis can be effectively treated with single dose oral therapies of *praziquantel* that are safe, inexpensive and required at periodic intervals [5]. Treatment is typically implemented through mass chemotherapy whereby the entire at-risk population is treated, as part of either school or community- based campaigns, referred to as mass drug administration (MDA).

The transmission intensity of Schistosomiasis is a function of parasitic worm load within a group of individuals, which can indirectly be quantified by the number of eggs that are excreted. Host heterogeneities in exposure and susceptibility to infection may lead to an aggregated distribution of worm burden across individuals [6]. For this reason, a few individuals would harbour large numbers of worms, whilst the majority of individuals are uninfected or only carry a low worm burden [6]. In addition, widely used diagnostic approaches for Schistosomiasis like the Kato-Katz technique for *S.mansoni* diagnosis fail to detect some infected individuals, particularly when only a single stool sample is examined and infection intensities are light [7]. Due to these two issues, often a large proportion of individuals are considered as “zero egg excretor” [6]. The standard Poisson distribution, which assumes equal mean and variance, commonly employed to model such count data, is inappropriate to fit observed egg counts since the variance of the counts is much larger than their mean, a case known as over-dispersion [8]. The use of negative binomial (NB) distribution has been proposed to model the extra-Poisson variation [9], and applications of NB in analysing helminth egg counts are many [8], [10].

Although NB models may be ideal for over-dispersion, they may not be suitable when data is zero-inflated. Other distributions like the hurdle models or zero inflated (ZI) or zero augmented models that may be more appropriate for modeling data with such excess zeros are reported [8]. These models can have more than one mode, including a mode at zero. ZI models attempt to account for excess zeros, i.e., zero inflation arises when one mechanism generates only zeros and the other process generates both zero and nonzero counts hence they can be expressed as a two-component mixture model where one component has a degenerate distribution at zero and the other is a count model [11]. ZI models estimate two equations, one for the count model and one for the excess zeros. ZI models assume that a proportion of individuals have no chance to be infected, as they are not exposed. In other words, there is a process which determines whether an individual is likely to be infected at all and a second process determining the number of excreted eggs among those who are at risk of infection. Zero inflated Poisson(ZIP) models assume that the number of excreted eggs follows a Poisson distribution. Zero-inflated negative binomial (ZINB) models assume that the number of worms among those who are at risk of infection has a negative binomial distribution [6].

ZI count data are common in a number of applications. Examples of data with too many zeros from various disciplines include agriculture, econometrics, patent applications, species abundance, medicine, and use of recreational facilities [8]. The zero-inflated Poisson(ZIP) regression models with an application to defects in manufacturing is described in [12], while zero-inflated binomial (ZIB) regression model with random effects into ZIP and ZIB models are defined in [13].

The idea for a hurdle model, a modified count model in which the two processes generating the zeros and the positives are not constrained to be the same, was developed in [14]. The two processes are modeled using a mixture of two models (i.e, two part or a hurdle model). The first part is a binary outcome model, and the second part is a truncated count model. Such a partition permits the interpretation that positive observations arise from crossing the zero hurdle or the zero threshold. The first part models the probability that the threshold is crossed, in our case thatan infection occurred. In principle, the threshold need not be at zero; it could be any value, and it need not be treated as known. The zero value has special appeal because in many situations it partitions the population into subpopulations in a meaningful way, one on infection status and the other for those infected it captures intensity. In contrast to the zero-inflated model, the zero and non-zero counts are separated in the hurdle model [15] which makes them very useful in inferential studies. Hurdle models are sometimes referred to as zero-altered models [16]. Zero-altered Poisson and negative binomial models are thus referred to, respectively, as ZAP and ZANB. They have also been termed overlapping models [17].

The application of hurdle or two part models in epidemiology has not been common so far. Use of ZI models have been reported. One such an application was in Cote d'Ivore, in which a ZINB model within a model-based geostatistics (MBG) framework for *S. mansoni* infection was applied [6]. This study showed that geostatistical ZI models produce more accurate maps of helminth infection intensity than the spatial negative binomial counterparts. However, to our knowledge, no hurdle or two part model has been applied in Schistosomiasis or geohelminth epidemiology.

This paper demonstrates the applications of hurdle models to helminth epidemiology (*S. haematobium*) and encourage its wider application in helminth disease control programmes. Its advantage is that it allows joint modeling of infection status and intensity. Although, a multinomial model maybe used [18]–[20], its limitation is that it involves stratifying egg counts, leading to a loss of information, whereas the negative binomial hurdle model approach makes full use of intensity data on a continuous scale, therefore, ideal to model latent infection intensity. In addition, hurdle models are robust when over-dispersion is present. In [8], it was concluded that the ZIP models were inadequate for the data as there was still evidence of over-dispersion. Moreover, the negative binomial hurdle model, which allows for over-dispersion and accommodates the presence of excess zeros through a two-part model has a natural epidemiological interpretation within the case study considered here.

## Materials and Methods

### Data

The data which motivated this work were collected in 2004 in Chikhwawa district, in the Lower Shire Valley-southern Malawi. This is a rural area whose population is mainly engaged in subsistence farming. This area lies between 100 and 300 m above sea level. The rainy season extends from December to March. Temperatures can rise up to 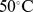 in months preceding rainy season. Malaria is known to be holoendemic [21].

Data were collected in eighteen villages, purposively selected from the control and intervention arms of a cluster randomized study design. There was only one round of treatment following community based and house to house approaches for mass drug administration (MDA). Over 90 percent of the eligible population were treated. All infected participants in non-intervention arm received appropriate treatment. After the follow-up assessment, both arms had mass treatment. In the study, polyparasitism was considered basing on the number of species an individual was hosting. The focus was on Hookworm, *S. mansoni*, *S. haematobium* and Ascaris. Polyparasitism is the epidemiology of multiple species parasite infections. Ten percent of the households were randomly selected from the villages for baseline survey using random number tables [22].

Subjects for geo-helminth survey were selected using a two stage-design. Briefly, at first stage, villages were selected, then at second stage, sample of households was listed and chosen. In the selected households all members aged one year and above were invited to participate. Consenting individuals had their demographic details completed and were given full body clinical examinations (except genitals for females) for chronic manifestations of human helminths. In addition they had anthropometric measurements taken and were asked to provide a single fresh stool and urine sample. All individuals (aged>1 year) were requested to provide a finger prick blood sample [22]. Further details are provided in [22].

### Laboratory procedures

Fresh stool samples were transported in a cooler box to the laboratory and processed within four hours of collection. A single Kato-Katz thick smear was prepared from each sample and immediately examined under a light microscope for parasite eggs (within 15–20 minutes). Standardized and quality controlled procedures were followed. Briefly 41.7 mg of sieved stool was placed on a microscope slide through a punched plastic template. Ova for each parasite observed were counted and expressed as eggs per gram (epg) of stool. Five percent of the slides were randomly selected for re-examination for quality control purposes [22].

Urine samples were processed on the day of collection. A measured volume (maximum 10 ml) was centrifuged at 300 rpm for five minutes. The sediment was then examined under a light microscope. The eggs seen were counted and the intensity of infection per 10 ml of urine accordingly determined. All those infected were treated with *praziquantel* at 40 mg/kg [22].

### Ethical approval

The study that collected data from Chikhwawa, Malawi received ethical clearance from Malawi's College of Medicine Research Ethics Committee (COMREC) [22]. Individual informed consent was orally obtained from each participant or (if they were aged<16) from one of their parents or a legal guardian. COMREC approved oral informed consent because the study was determined to be of minimal risk. The consent process was a four stage process. First stage, oral informed consent was obtained at the traditional authority (TA) level. Second stage, at village head level and third stage at the household level from the head of the household and fourth at individual level from each individual in the household (if applicable) else from parent/guardian if an individual was aged<16. Registers were kept for documentation whereby, for each individual in the selected household, a column was kept to indicate whether an individual had orally consented to participate in the study or not.

### Statistical methods

Various statistical models have been developed to model helminths disease burden as reviewed in the introduction. For purposes of this paper, we assumed a negative binomial logit hurdle (NBLH) model for joint analysis of infection prevalence and intensity of Schistosomiasis hematobium in Malawi. Following on [11], a NBLH model can be written as:
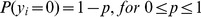
(1)

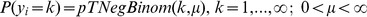
(2)where 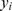 are observed counts taking values 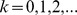 for each individual 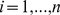. The probability of infection is 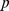, such that 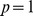 indicates there are no zero counts and the model reduces to a truncated Negative Binomial distribution (TNegBinom); while 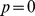 means there are no infections. The observed counts are modelled by assuming two processes:
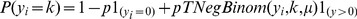
(3)The first is assumed to model the infection prevalence (first hurdle) and the other the intensity of infection (second hurdle). The first hurdle assumes a binary outcome defining whether an individual is infected or not. This is modeled as a logit regression for a given set of risk factors 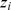. After determining infection status we are interested in analyzing the number of eggs - as a measure of intensity of infection, which is defined by the second hurdle. We model the second hurdle as a negative binomial regression model for a given set of risk factors 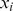. The NB model is suited for count data with over-dispersion. In many cases, the same risk factors are used in the logit and count regression models, i.e. 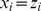. The two regression models, incorporating the risk factors, are given by:
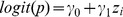
(4)

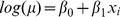
(5)The model parameters 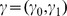 and 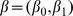 are estimated using maximum likelihood estimation in which the likelihoods (or log-likelihoods) are maximized separately.

The covariates included in the model are given in Table 1. Age and polyparasitsm were fitted as continuous variables, while sex, education levels, village type, fishing, gardening and occpuation were entered in the model as categorical variables, with the first category of each variable selected as the reference group. For both parts of the model we used the same set of covariates. We also fitted a number of count models, with the Poisson as the null model, for comparison and evaluated the number of zeros each model correctly predicts. We also compared model fit using AIC and zero capturing. A difference of 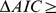 10 indicates the model with the smallest AIC is superior to others. Furthermore, deviance residuals were assessed for spatial correlation using variogram and were subsequently mapped using kriging to depict spatial variation in risk. Statistical model fitting was carried out using Political Science Computational Laboratory (PSCL) package [23] in R statistical software (The R Foundation for Statistical Computing, Version 2.14.0). Variogram analysis and kriging were implemented in geoR [24].

**Table 1:** Characteristics for individuals who had *S. haematobium*.

Variable	Mean(Std. Dev)	Number(%)
*Outcome*		
S. haematobium		233 (14.2)
*Age (years)*	32.36 (22.79)	
*Sex*		
Female		909 (55.36)
Male		733 (44.64)
*Education level*		
None		745 (45.37)
Primary		850 (51.77)
Secondary		47 (2.86)
*Village type*		
Intervention		831 (50.61)
Control		811 (49.39)
*Fishing*		
Yes		1,421 (86.54)
No		221 (13.46)
*Garden*		
Yes		960 (58.47)
No		682 (41.53)
*Occupation*		
Farmer		733 (44.64)
Other		909 (55.36)
*Polyparasitism*		
None		807 (49.15)
One species		594 (36.18)
Two species		200 (12.18)
Three species or more		41 (2.49)

## Results

### Characteristics of participants

Table 1 gives summary statistics for study participants. The study had 1642 participants of which 55.4 % were female. The mean age (years) of 32.4 (standard deviation: 22.8). Of these, 324 had hookworm representing 19.7 % of sample population, 71 of these had *S. mansoni* representing 4.3 % and 233 had *S. haematobium* representing a prevalence of 14.2 %.

**Figure 1:**
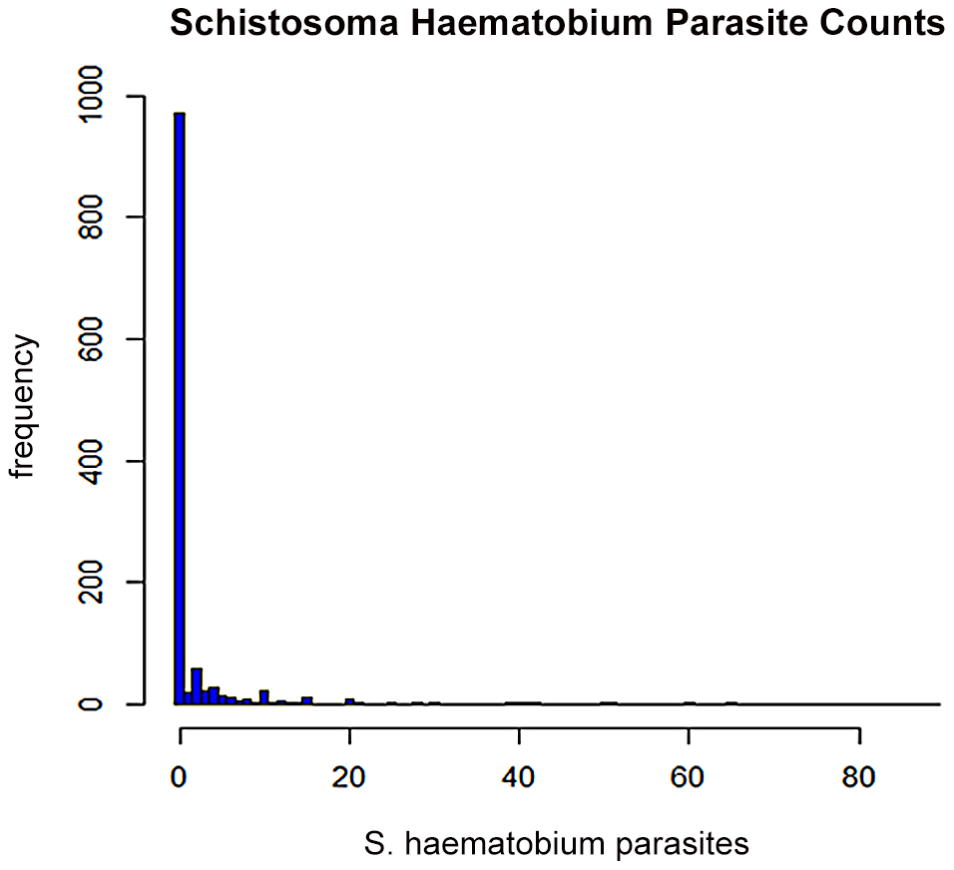
The distribution of parasite counts of *S. haematobium*, measured as eggs/10 ml, in individual participants.

Figure 1 shows that a large proportion of individuals i.e. 85.8 % were “zero egg excretors” hence the data were inflated with zeros. The likelihood ratio test for overdispersion between Poisson and negative binomial at 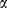 = 0.05 showed a critical value test statistic = 2.7 with a 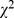 test statistic = 10606.5, p-value<0.001. Indeed, there was overwhelming evidence of overdispersion. This was confirmed by the presence of excess zeros (Figure 1).

### Model comparison

Using the AIC and zero capturing, the predicted counts using the NBLH indicate a closer fit with the observed values. In Table 2, AIC results show that the NBLH offers a better fit compared to using Poisson Logit Hurdle (PLH) or a negative binomial (AIC = 3,482 for NBLH; AIC = 6,854 for PLH and AIC = 3,576 for NB respectively). The AIC further showed a difference of 10,700 for the NBLH compared to the Poisson and a difference of 19 comparing NBLH with ZINB, thus NBLH is superior among all competing models. With regards to zero capturing, the Poisson model was again not appropriate as it could only capture 515 of the zeros whereas the NB-Zero adjusted based models were much better in capturing the zero counts. The NBLH model captured 971 zeros which were equal to the observed (Table 3). Since NB logit hurdle model offered the best fit to zero inflated helminth data in terms of the AIC (minimum value for all the models fitted) as well as true zero count capturing, it therefore became a natural choice for fitting a final model to model helminth infection intensity and determination of factors that foster infections.

**Table 2:** Model comparison using Akaike Information Criterion (AIC).

	Poisson	Neg. Bin.	ZIP	ZINB	PLH	NBLH
**AIC**	14,182	3, 576	6, 854	3, 484	6, 854	**3, 482**
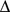 **AIC**	0	10,606	7,328	10,681	7,328	10,700

Models compared are Poisson, Negative Binomial (NB), Zero-Inflated Poisson (ZIP), Zero-Inflated Negative Binomial (ZINB), Poisson Logit Hurdle (PLH) and Negative Binomial Logit Hurdle (NBLH).

**Table 3:** Zero count capturing.

Observed	Poisson	Neg. Bin	ZIP	ZINB	PLH	NBLH
971	515	968	970	969	970	**971**

Models compared are Poisson, Negative Binomial (NB), Zero-Inflated Poisson (ZIP), Zero-Inflated Negative Binomial (ZINB), Poisson Logit Hurdle (PLH) and Negative Binomial Logit Hurdle (NBLH).

### Fixed effects of infection probability

Table 4 provides estimates for the fixed effects. The probability of infection was found to be associated with age (Odds Ratio [OR] = 0.97, 95 % Confidence Interval [CI]: 0.96–0.99), the risk of infection was decreasing with age. This assumed a linear relationship with age; 6 years being the baseline age. The risk of infection was low in males than in females (OR = 0.61, 95 % CI: 0.41–0.89). The association between risk of infection with education at both primary level (OR = 1.18, 95 % CI: 0.81–1.71) and secondary level (OR = 1.37, 95 % CI: 0.41–4.60) relative to those with no education was not significant (p-value = 0.62). Infection probability was found to be associated with village type; whether one was in the intervention area or control area (OR = 0.38, 95 % CI: 0.26–0.54, p-value<0.001). Those in the intervention area were at a reduced chance of infection relative to those in control area. We observed a negative association between infection probability and fishing (OR = 0.73, 95 % CI: 0.44–1.20) though not significant; contrary to the expectation. Working in the garden was observed not to be significant albeit it was positive (OR = 1.34, 95 % CI: 0.90–1.99). Again, occupation (farmer/other) showed a negative association with infection probability though with marginal significance (OR = 0.61, 95 % CI: 0.35–1.06) with a p-value = 0.17. We also noted that chances of infection were increasing with number of parasite species an individual was hosting (Table 4) (OR = 7.30, 95 % CI:5.56–9.59).

**Table 4:** Fixed effects estimates for negative binomial logit hurdle model for *S. haematobium*.

	Infection Probability		Infection Intensity	
	OR	95 % CI	RR	95 % CI
*Intercept*	0.13	(0.06,0.29)	11.72	(5.70,24.08)
*Age*	0.97	(0.96,0.99)	0.96	(0.95,0.98)
*Sex:*				
Female	1.00		1.00	
Male	0.61	(0.41,0.89)	1.03	(0.72,1.47)
*Education level:*				
None	1.00		1.00	
Primary	1.18	(0.81,1.71)	1.54	(1.08,2.19)
Secondary	1.37	(0.41,4.60)	0.34	(0.11,1.06)
*Village type:*				
Control	1.00		1.00	
Intervention	0.38	(0.26,0.54)	0.81	(0.58,1.13)
*Fishing:*				
No	1.00		1.00	
Yes	0.73	(0.44,1.20)	0.68	(0.45,1.03)
*Garden:*				
No	1.00		1.00	
Yes	1.34	(0.90,1.99)	1.21	(0.82,1.81)
*Occupation:*				
Other	1.00		1.00	
Farmer	0.61	(0.35,1.06)	1.83	(1.16,2.91)
*Polyparasitism*	7.30	(5.56,9.59)	0.87	(0.70,1.08)

### Fixed effects of infection intensity

From Table 4, it was observed that infection intensity reduced with an increase in age (Relative Risk [RR] = 0.96, 95 % CI: 0.95–0.98). Similar to infection prevalence, a linear relationship was assumed between infection intensity and age. There was no difference of infection intensity between males and females (RR = 1.03, 95 % CI: 0.72–1.47). Primary school children showed a high infection intensity relative to those that are in pre-school level (RR = 1.54, 95 % CI: 1.08–2.19) whereas those in secondary level showed a reduced infection intensity (RR = 0.34, 95 % CI: 0.11–1.06) though not significant. There was a reduced risk for those in intervention area relative to those in the control area, though, not significant (RR = 0.81, 95 % CI: 0.58–1.13). A positive association was also observed between those who did fishing in Shire river relative to those who did not fish (Table 4). We observed an increased infection intensity in those working in the gardens relative to those who did not (RR = 1.21, 95 % CI: 0.82–1.81), albeit not significant and also increased infection intensity for farmers compared to non-farmers (RR = 1.83, 95 % CI: 1.16–2.91).

### Residual spatial variation

**Figure 2:**
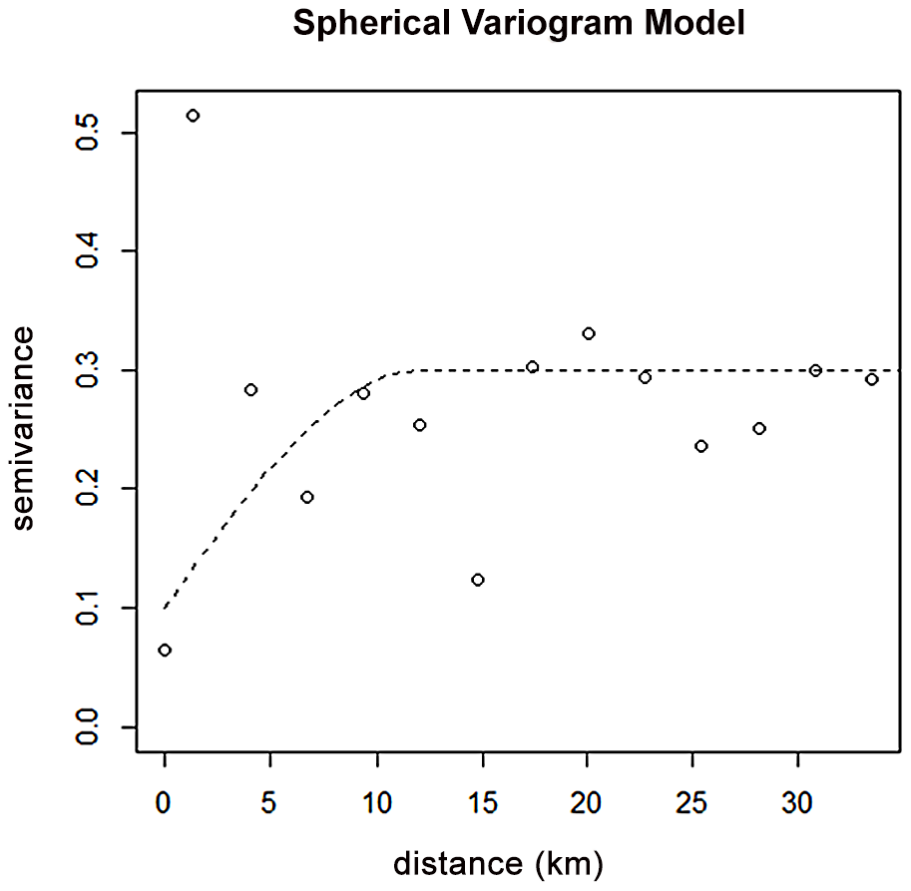
Spherical variogram plot based on the deviance residuals. **The sill is at 0.3 at range 0f 10 kms.**

**Figure 3:**
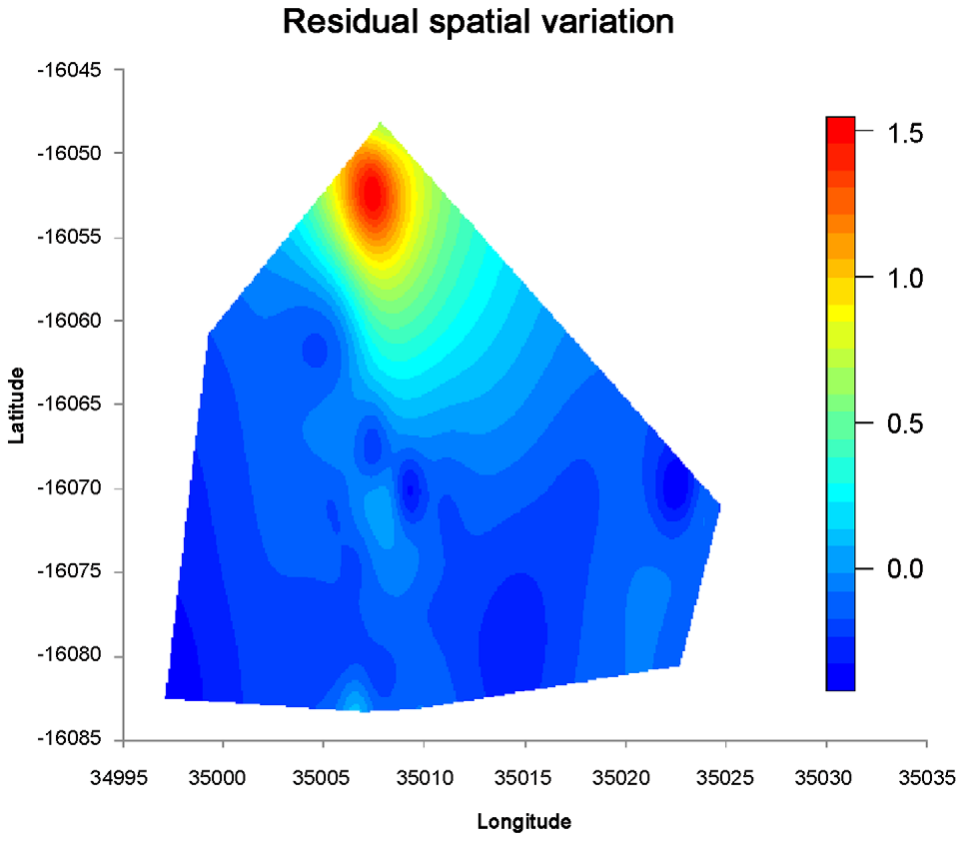
Estimated residual spatial effects of *S. heamatobium* infection, given in log odds ratios.

Estimating the continuous surface using variogram analysis and kriging, spatial patterns in the residuals were observed and subsequently mapped. There was some degree of spatial dependence in residuals distribution across the study area, as evidenced by the spherical model (Figure 2). The magnitude of spatial correlation decreased with separation distance until at distance of 10 km. The predicted spatial surface, in Figure 3, showed a relatively increased risk of infection in the northern part of the study area compared to other areas. Low risk areas were in the southern parts, more especially in the south-eastern part of the study region (Figure 3).

## Discussion

The current study found a prevalence of 14.2 % for *S. haematobium* in Chikhwawa district. This prevalence was well below national estimates, which a previous study in Malawi indicated to be between 40 and 50 % [25]. The finding serves to highlight the fact that Schistosomiasis infections are highly localised and that nationwide surveys tend to overlook the focus of heterogeneity of infection. Indeed, in a study conducted in the northern lakeshore area [26], school children from four schools screened for Schistosomiasis reported a wide range of prevalence: 5 %–57 % of *S. haematobium* infection. A national survey, representative of all school children in the country, and undertaken just before the rainy season, showed far lower levels of 7 % for *S. haematobium* [25].

We used robust, contemporary statistical methods in a two part application to analyse risk factors for *S. haematobium* infection intensity and prevalence. This resulted in estimates of parasitic infection prevalence and intensity that could be used in control programme planning by channeling resources to areas with a known high disease burden. In this study we have looked at the intensity and prevalence of *S. haematobium* in relation to factors such as age, sex, education level, village type, fishing in Shire river, working in gardens, occupation and polyparasitism. Polyparasitism is the epidemiology of multiple species parasite infections. In the study, polyparasitism was based on the number of species an individual was hosting. The focus was on Hookworm, *S. mansoni*, *S. haematobium* and ascaris. The study confirms the critical importance of ascertaining the infection intensity.

We found that *S. haematobium* infection intensity reduced with age, this confirms what previous studies found. In common intestinal helminths such as *Ascaris lumbricoides* (large roundworms) and *Trichuris trichiura* (whipworm) and also Schistosomiasis, children are more heavily affected and infected than adults [27]. Several other studies have reported that school-aged children show high infection intensity and prevalence [25], [28], [29]. Fishing in Shire river and working in gardens along the river were potential risk factors for exposure to schistosomes and subsequent infection because transmission requires contact with the aquatic habitat of intermediate host snails [30]. This is in line with results from a study that was conducted in western Africa [20], that contact with water bodies that are a habitat for intermediate host snails is one of the main risk factors. Results showed low probability of infection for males compared to females. This could be explained by a number of factors including that Malawi being an agriculture based economy, and that mainly agricultural activities are carried out by females, hence they are more exposed to risk factors such as working in gardens and farming. Schistosomiasis is water dependent disease and the incidence is usually more amongst people who constantly get into contact with the schistosome infected waters through activities such as farming, fishing, swimming and washing [30].

Results from the study showed that individuals who had received chemotherapy cure for helminth showed reduced risk of infection as well as infection intensity as compared to those in the control area. Studies have shown that MDA significantly reduces Schistosomiasis infection [31], [32]. Evidence has shown that, following chemotherapeutic cure of *S. mansoni* or *S. haematobium* infection, older individuals display a resistance to re-infection in comparison to younger children [33]. Therefore there is need to channel integrated control and interventions for helminths to areas with diseases burden in order to reduce and/or eradicate the infections - more especially towards school age children. Several studies have shown that having one infection, is a risk factor for having other infections [34]. It is conceivable that the first parasite that establishes an infection may modulate the immune response in such a way that it makes it easier for the next [22].

Worthy noting were differences that existed in associations between infection probability and infection intensity. For gender, males had a reduced risk of infection as compared to females (negative association) but high infection intensity (positive association). This could possibly be explained by the fact that women were mostly involved in agricultural activities there by being more exposed. Also for those infected, many studies find that men visit public health care facilities much less frequently than do women [35] hence the high intensity. Poly-parasitism was positively associated with infection probability but had a negative association with infection intensity. This could be explained by the fact that having other parasites increases the chance of the body being susceptible to new parasite infections [34]. Again, secondary level of education had a positive association with infection probability but showed a negative association with infection intensity. This finding could be explained by the fact that an increase in education level corresponds to increase in age which comes with increased risk-behaviour of older school children who frequently contact schistosome-infested water for both domestic and livestock purposes relative to younger children [36] hence increased infection prevalence. At the same time, an increase in education may correspond to increased awareness and access to treatment [37] by this group hence reduced infection intensity. Those with the highest level of education, through high school, have showed the lowest mean infection intensity [37]. Being a farmer had a negative association with probability of infection and a positive association with infection intensity. The finding was in line with what was reported in [37]; farmers showed the highest levels of Schistosomiasis infection among occupational groups. Both education and occupation are proxies for the nature and intensity of water contact [37]. Individuals become infected by prolonged contact (like irrigating farm, bathing, washing or swimming) with fresh water containing free-swimming Cercariae [30].

We believe that the apparent dominance of agricultural, socio-economic and demographic factors in determining *S. haematobium* infection risk in the villages carries important implications for disease surveillance and control strategies. Prevalence of *S. haematobium* was highly associated with age of an individual as well as working in the garden and also number of parasites an individual hosted. Furthermore, *S. haematobium* infection intensity was associated with gender, education level, garden, occupation and village type (intervention). Cercariae control control through environmental modifications and strategies involving socio-economic status improvement and MDA may be more promising approaches to disease control in this setting.

Finally, zero adjusted methods represents a key advance in the epidemiological analysis of helminth disease data inflated with zeros. There are an increasing number of examples in the published literature where two part methods are being used for zero inflated data for helminths disease's control planning and implementation programmes [38], [39]. Ease of implementation and straightforward interpretation of the components and its direct link with the observed data, makes the negative binomial logit hurdle model definitely a valuable alternative for researchers analysing zero-inflated count data for helminths.
